# Overlap Arrhythmia Syndromes Resulting from Multiple Genetic Variations Studied in Human Induced Pluripotent Stem Cell-Derived Cardiomyocytes

**DOI:** 10.3390/ijms22137108

**Published:** 2021-07-01

**Authors:** Jacqueline A. Treat, Ryan Pfeiffer, Hector Barajas-Martinez, Robert J. Goodrow, Corina Bot, Rodolfo J. Haedo, Ronald Knox, Jonathan M. Cordeiro

**Affiliations:** 1 Department of Experimental Cardiology, Masonic Medical Research Institute, Utica, NY 13501, USA; incredimomj@hotmail.com (J.A.T.); pfeifferr@mmri.edu (R.P.); goodrowr@mmri.edu (R.J.G.); 2 Department of Cardiovascular Research, Lankenau Institute for Medical Research, Wynnewood, PA 19096, USA; barajasmartinezh@mlhs.org; 3 Nanion Technologies, 1 Naylon Ave. Suite C, Livingston, NJ 07039, USA; corina.bot@gmail.com (C.B.); Rodolfo.Haedo@naniontech.com (R.J.H.); ronald.knox@naniontech.com (R.K.)

**Keywords:** action potentials, depolarization, electrophysiology, sodium current, stem cells

## Abstract

Human induced pluripotent stem cell-derived cardiomyocytes (hiPSC-CMs) are used for genetic models of cardiac diseases. We report an arrhythmia syndrome consisting of Early Repolarization Syndrome (ERS) and Short QT Syndrome (SQTS). The index patient (MMRL1215) developed arrhythmia-mediated syncope after electrocution and was found to carry six mutations. Functional alterations resulting from these mutations were examined in patient-derived hiPSC-CMs. Electrophysiological recordings were made in hiPSC-CMs from MMRL1215 and healthy controls. ECG analysis of the index patient showed slurring of the QRS complex and QTc = 326 ms. Action potential (AP) recordings from MMRL1215 myocytes showed slower spontaneous activity and AP duration was shorter. Field potential recordings from MMRL1215 hiPSC-CMs lack a “pseudo” QRS complex suggesting reduced inward current(s). Voltage clamp analysis of I_Ca_ showed no difference in the magnitude of current. Measurements of I_Na_ reveal a 60% reduction in I_Na_ density in MMRL1215 hiPSC-CMs. Steady inactivation and recovery of I_Na_ was unaffected. mRNA analysis revealed ANK2 and SCN5A are significantly reduced in hiPSC-CM derived from MMRL1215, consistent with electrophysiological recordings. The polygenic cause of ERS/SQTS phenotype is likely due to a loss of I_Na_ due to a mutation in *PKP2* coupled with and a gain of function in I_K,ATP_ due to a mutation in *ABCC9*.

## 1. Introduction

Induced pluripotent stem cells derived from humans (hiPSCs) are utilized for investigations of cardiac genetic diseases affecting ion channels and/or structural proteins [1,2]. Using fibroblasts obtained from the patient, the fibroblasts can be de-differentiated into iPSCs and then directed to the cardiac lineage to allow systematic evaluation of the effects of these mutation(s) on ion channel function. Often the clinical phenotype observed in the patient is not due to a single mutation but may be the result of multiple genetic variations. Numerous cardiac diseases have been studied in hiPSC models and these models were able to capture the electrophysiological alterations observed in the clinic [3,4,5]. 

Many arrhythmias occur in the atria or ventricle and appear in the absence of structural defects. For example, short QT syndrome (SQTS) and early repolarization syndrome (ERS) are conditions linked to variations in ion channels [6,7,8]. SQTS is characterized by a QTc < 330 ms and as many as six different genes have been linked to SQTS [9]. ERS (sometimes referred to as J-wave syndrome [10]) typically appears as a slurring or notch in the ST segment and nine genes have been associated with this syndrome [11]. The QT interval is governed by the ventricular action potential duration which depends on the balance of currents during the repolarization phase of the action potential. In SQTS, a reduction in inward currents like I_Ca_ or increases in outward repolarizing currents such as I_Kr_, I_Ks_ or I_K1_ leads to shortening of the action potential duration and QT interval [9,12]. In contrast, the slurring or notch in the ST segment observed in ERS patients suggests changes in phase one and phase two of the cardiac action potential secondary to an increase in I_to_ or decreases in I_Na_ or I_Ca_ [13]. 

Some cardiac channelopathies such as SQTS have shown a clear structure–function alteration due to a single mutation in one ion channel [14,15,16] whereas as other investigations show that multiple genetic variations may be involved [17,18]. Interestingly, many of these genetic variations are in proteins which are not cardiac ion channels. For example, GWAS analysis has found a high association between Brugada Syndrome (BrS) and variations in the neuronal Na^+^ channel *SCN10A* [19] yet little to no Nav1.8 protein has been measured in cardiac tissue [20]. Similarly, variations in *PXDNL* which encodes a peroxidasin-like protein, [21] have been identified in 45% of patients with cardiac arrhythmia syndrome [18]. These studies further highlight the fact that multiple genes are involved in various cardiac arrhythmia syndromes. 

In this study, we identified an individual that presented with an initial diagnosis of ERS and SQTS who exhibited multiple episodes of syncope following severe electrocution. Utilizing hiPSC- cardiomyocytes derived from the patient, medium throughput extracellular field potential analysis revealed a complete absence of ‘pseudo’ QRS complex suggesting a dramatic loss of inward currents responsible for initiating excitation. Interestingly, genetic analysis showed that the patient carried multiple variations in ion channel and structural proteins but none of these mutations were in the alpha subunit of genes responsible for Na^+^ or Ca^2+^ channels. This disconnect was systematically evaluated using hiPSC-CMs derived from the patient. 

## 2. Results

**Figure 1:**
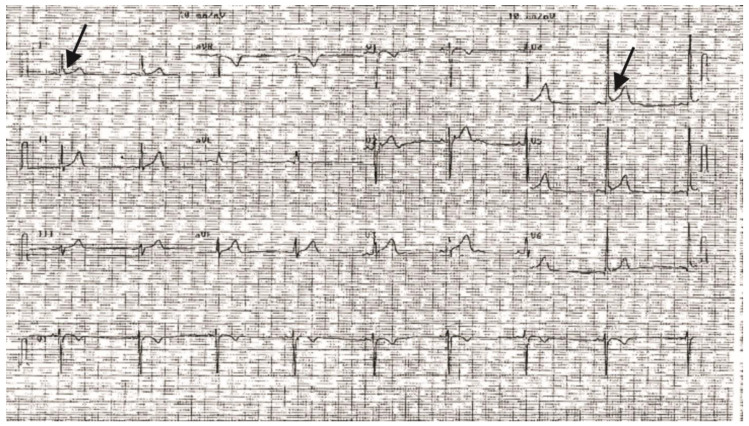
Representative ECG of MMRL1215 illustrating Early Repolarization Syndrome phenotype. A slurring of the QRS complex is apparent in multiple leads (denoted by the arrows). In addition, a QTc = 326 ms was noted.

The index patient presented to the physician because of repeated episodes of syncope that developed several weeks after the patient was electrocuted while driving a forklift. Patient was a 27-year-old Caucasian male who was found unconscious after receiving the high voltage electrical shock. Analysis of the ECG revealed an ERS phenotype (Figure 1) coupled with a QT interval of 326 ms suggesting a secondary diagnosis of SQTS. Patient was inducible following programmed electrical stimulation with triple premature 400/240/200/20 prior to procainimide challenge. After procainimide administration, the patient was very easily inducible which required cardioversion.

We performed NextGen sequencing of the index patient. Genetic analysis of the index patient revealed six heterozygous exonic mutations. Of the exonic mutations found, three were associated with ion channels and three of the mutations were predicted to be damaging by Sift analysis. The *ANK2* gene was mutated from a T to A substitution at position 10901 in exon 41, resulting in an amino acid change from valine to aspartate at position 3634 (V1085D). The *ABCC9* gene was mutated from C to T at position 1987 in exon 14 resulting in an amino acid change from arginine to cysteine at position 663 (R663C). The *PKP2* gene was mutated from a G to A at position 76 in exon 1 resulting in an amino acid change from an aspartate to an asparagine at position 26 (D26N). Mutations and polymorphisms of the proband are shown in Table 1. 

**Figure 2:**
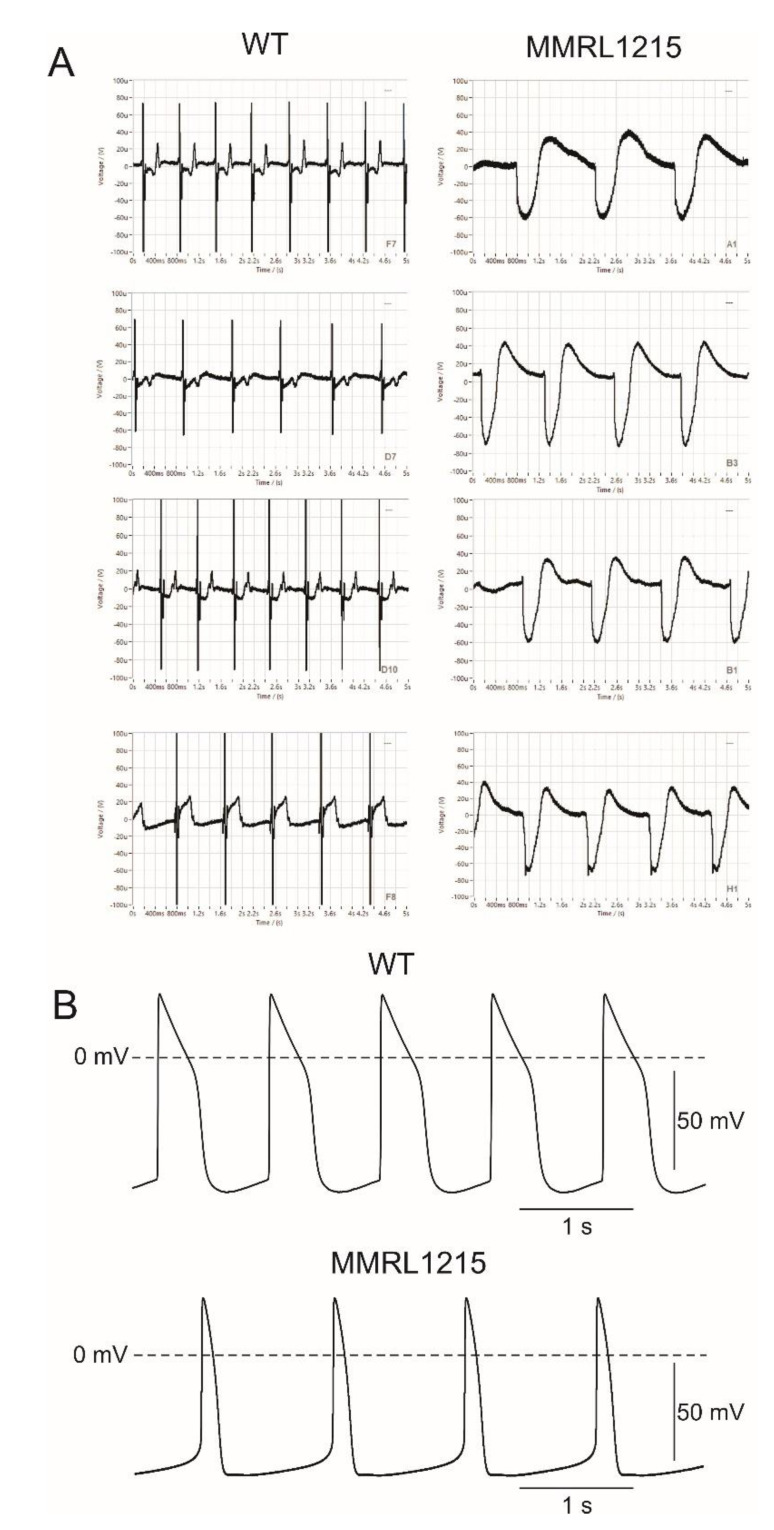
(**A**): Extracellular field potential (EFP) signals recorded in WT (left side) and MMRL1215 hiPSC-CMs (right side). Compared to WT hiPSC, myocytes derived from MMRL1215 showed a complete lack of a pseudo QRS complex, suggesting a major reduction in inward current(s). Panel (**B**): Action potential (AP) recordings obtained from WT and MMRL1215 monolayers. MMRL1215 hiPSC-CMs had a shorter action potential duration compared to WT.

To determine the functional effects of these mutations/variations, we recorded extracellular field potentials (EFPs) from normal (WT) hiPSC-CMs and hiPSC-CMs derived from patient MMRL1215. Cells were plated on the microelectrode array at ~75,000 cells per plate and EFP recordings were made. Figure 2A shows the EFP recordings from 4 representative WT and MMRL1215 wells. The field potential recordings from WT hiPSC-CMs showed a ‘pseudo’ QRS complex and T wave, suggesting a normal activation–recovery cycle. In contrast, EFP recordings from MMRL1215 hiPSC-CMs showed no distinct QRS complex. In addition, the spontaneous beating frequency was slower in recordings obtained from MMRL1215 with a rate of 64.8 ± 10.2 bpm for WT and 48.0 ± 10.8 bpm for MMRL1215. The absence of a pronounced QRS complex coupled with the slower beating rate would suggest multiple ion channels are affected resulting in the complexed changes observed in the field potential recordings. We next recorded action potentials from WT and MMRL1215 hiPSC-CM monolayers (Figure 2B). MMRL1215 myocytes showed a slower beating rate and had a shorter APD compared to WT. The shorter APD may be the result of a gain of function mutations in *KCNA5* and/or *ABCC9* present in the patient (Table 1). The *ABCC9* gene encodes the sulfonylurea receptor 2 (SUR2) protein which links with Kir6.1 and/or Kir6.2 to form a functional ATP-sensitive K^+^ channel [22]. These channels are closed under normoxic conditions but activate when there is a decrease in intracellular ATP levels [23]. We wondered if the mutation in *ABCC9* altered the function of the channel resulting in these channels opening in under normoxic conditions in MMRL1215 myocytes. We next tested the effects of 10 µM glybenclamide (an inhibitor of ATP-sensitive K^+^ channels) on APs recorded from MMRL1215. Application of glybenclamide resulted in a prolongation of the APD, suggesting these channels are open under normoxic conditions. Table 2 and Table 3 summarize the APs recorded from WT and MMRL1215 hiPSC-CM monolayers and the response to glibenclamide. 

**Figure 3:**
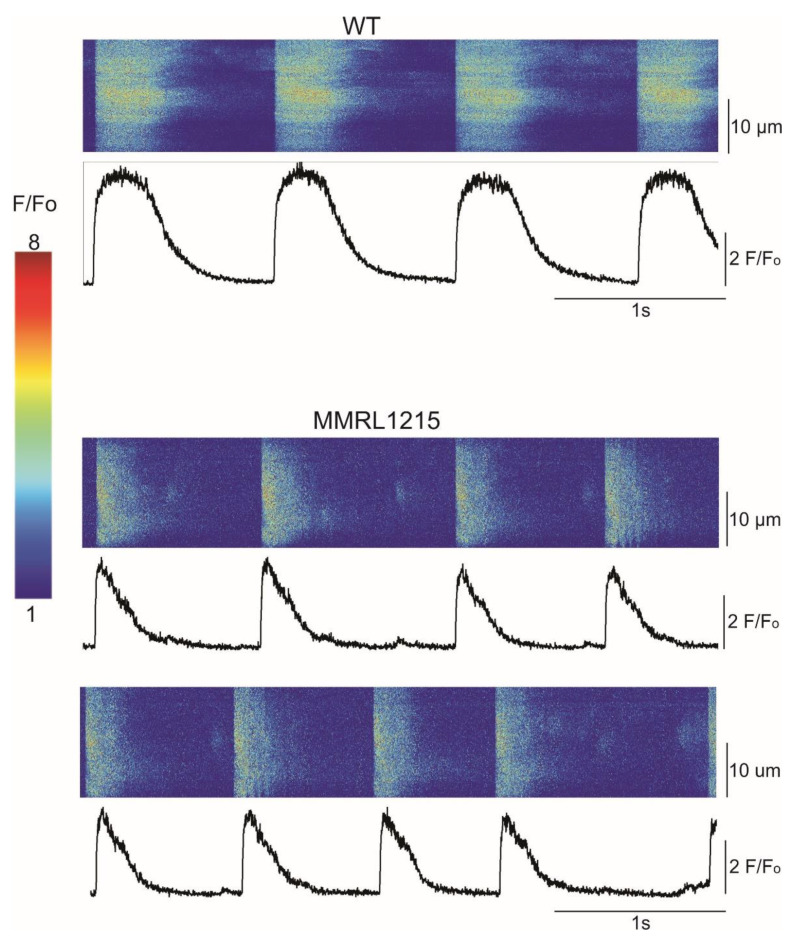
Line scans recorded from WT and MMRL1215 hiPSC-CMs showing spontaneous Ca^2+^ transients. Fluorescence intensity was much lower in MMRL 1215, and in some myocytes the spontaneous activity was episodic in nature.

Since EFP and AP recordings revealed differences in the electrophysiology, we speculated that Ca^2+^ transients would also be altered. Next we recorded calcium transients in WT hiPSC-CMs and hiPSC-CMs derived from patient MMRL1215 (Figure 3). The Ca^2+^ dye fluo-4 was loaded into hiPSC-CMs and Ca^2+^ transients were recorded from spontaneously beating cells. Compared to WT recordings, Ca^2+^ transients from patient 1215 showed lower fluorescence intensity (Table 4).

**Figure 4:**
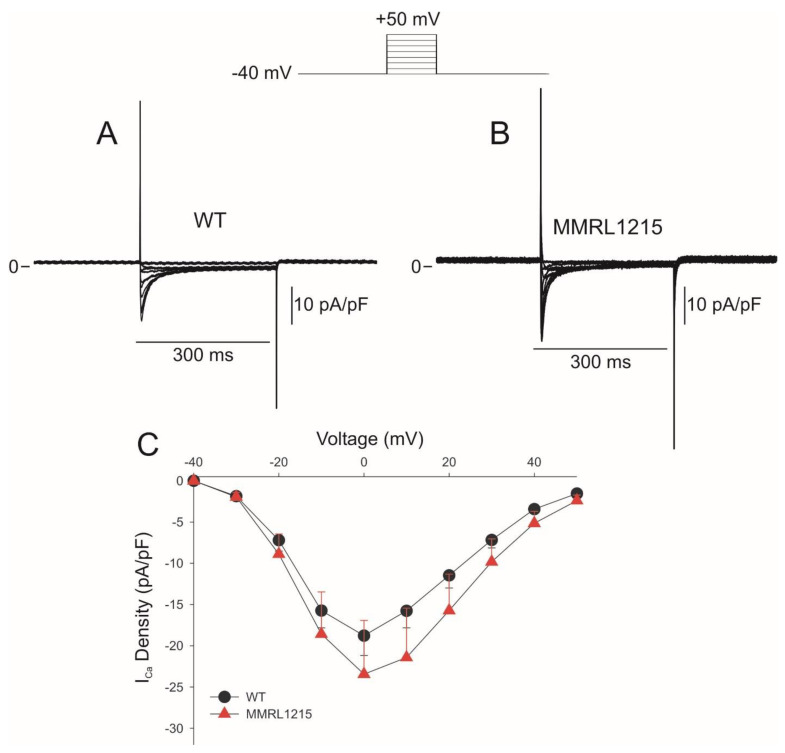
Representative traces showing I_Ca_ recorded from WT *(n* = 11) and MMRL1215 *(n* = 9) (**A**,**B**). Ca^2+^ currents were recorded during a 300 ms step depolarization from −40 to +50mV. (**C**) Current-voltage relationship for I_Ca_.

The lower fluorescence intensity in 1215 hiPSC-CMs may indicate that there is less intracellular Ca^2+^ during an action potential secondary to a shorter AP duration [24]. Alternatively, the EFP recordings suggested that inward current(s) were altered and, therefore, the density of I_Ca_ may be lower. We next performed patch clamp experiments to measure inward I_Ca_ from WT and MMRL1215 hiPSC myocytes (Figure 4). I_Ca_ was elicited by application of 300 ms step depolarizations from −40 to +50 mV and extended protocols were avoided to prevent current rundown. Peak I_Ca_ density was −18.8 ± 2.5 pA/pF for WT and −23.4 ± 6.5 pA/pF for MMRL1215. The summarized data on I_Ca_ current density in WT and MMRL1215 is shown in Figure 4C and no difference in current density was observed. 

**Figure 5:**
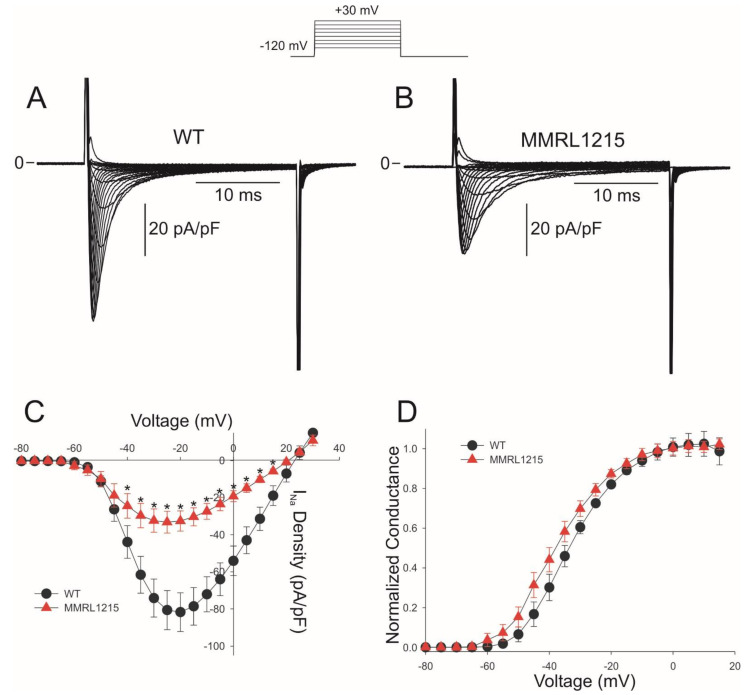
Representative whole cell I_Na_ recordings from WT and MMRL1215 hiPSC-CMs (**A**,**B**). Current recordings were obtained at test potentials between −80 and 30 mV (protocol at top of figure). (**C**): I–V relation for WT (*n* = 18) and MMRL1215 (*n* = 25) myocytes. (**D**): Steady-state activation curves for WT and MMRL1215 myocytes. Data were normalized and plotted against their test potential. * Statistically significant from WT, *p* < 0.05.

As mentioned previously, the absence of a QRS complex in MMRL1215 hiPSC-CMs would suggest that inward currents are greatly reduced compared to WT. We next determined if inward I_Na_ measured from hiPSC-CMs derived from the proband had a lower density compared to WT. Recordings were made from myocytes by manual patch clamp techniques in 40 mM external Na^+^ to ensure voltage control and representative I_Na_ traces are shown (Figure 5A,B). Analysis of the current–voltage (I–V) relation showed that MMRL1215 had a decreased I_Na_ density compared to WT myocytes (Figure 5C). Peak I_Na_ density was −80.6 ± 10.49 pA/pF for WT and −32.58 ± 6.28 pA/pF for MMRL1215. We also determined if this reduction in peak I_Na_ was due to changes in steady-state activation. Maximum conductance was obtained from the I–V curve and the ratio of current to the electromotive potential was calculated. A Boltzmann function was then fit to the data. Analysis of steady-state activation curves revealed half-activation voltages (V1/2) of −33.13 ± 0.39 mV, k = 8.13 ± 0.35 for WT and −37.61 ± 0.41 mV, k = 8.92 ± 0.36 for MMRL1215.

**Figure 6:**
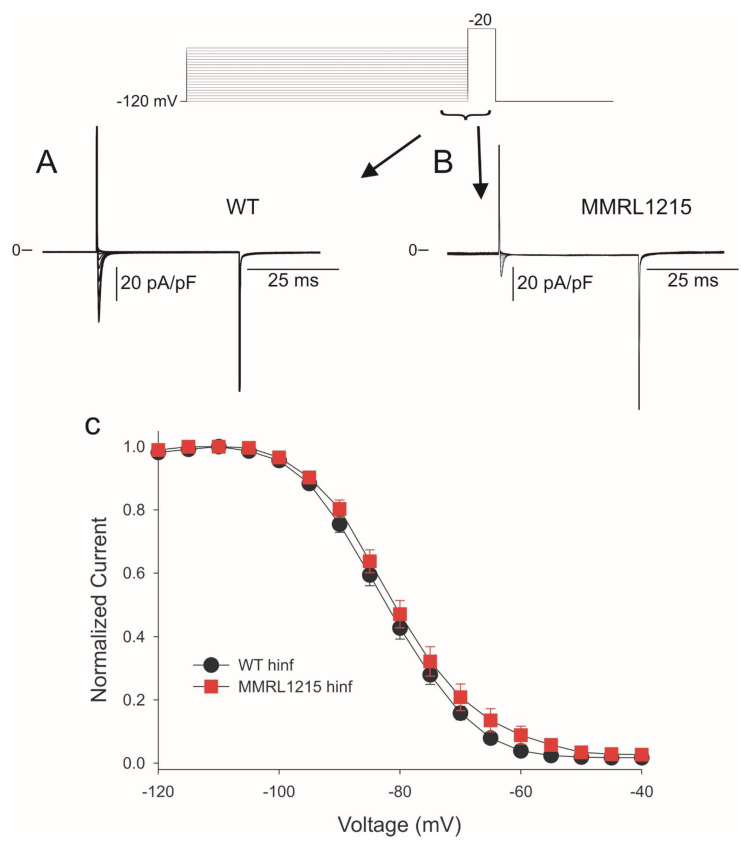
Representative steady-state inactivation recordings from WT and MMRL1215 myocytes (**A**,**B**) in response to the voltage clamp protocol (top of the figure). The steady state-inactivation relation for hiPSC-CMs (**C**) yielded similar mid-inactivation potential for WT (*n* = 25) and MMRL1215 (*n* = 29) cells.

The differences in I_Na_ density between WT and MMRL1215 may be the result of differences in Na^+^ channel availability at any given voltage. Steady-state inactivation was next evaluated in WT and MMRL1215 myocytes using a standard pre-pulse test pulse voltage clamp protocol. After application of a 500 ms pre-pulse, peak current was normalized to the maximum current and plotted as a function of the pre-pulse voltage. A Boltzmann function was then fit to the data. Figure 6 shows representative traces recorded from a WT (Panel A) and MMRL1215 myocyte (Panel B). The mid-inactivation potential was not significantly different in WT vs. MMRL1215 myocytes (Panel C) with V1/2 of −82.3 ± 0.21 mV, k = 6.79 ± 0.18 for WT and −81.1 ± 0.23 mV, k = 7.15 ± 0.21 for MMRL1215 suggesting decreased peak I_Na_ in MMRL1215 was not due to differences in this parameter.

**Figure 7:**
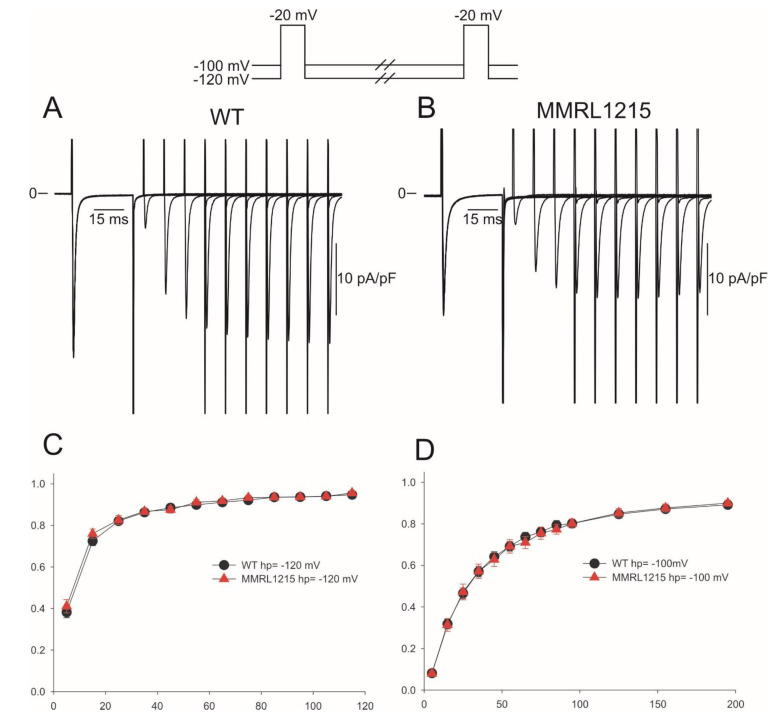
Representative recovery from inactivation traces recorded from WT and MMRL1215 myocytes (**A**,**B**). Recovery was measured using two identical voltage clamp steps to −20 mV from a holding potential of either −120 mV or −100 mV separated by selected time intervals. (**C**): Mean data showing recovery time-course of I_Na_ recorded at −120 mV for WT (*n* = 24) and MMRL1215 (*n* = 26). (**D**): Recovery time-course of I_Na_ recorded at −100 mV for WT (*n* = 16) and MMRL1215 (*n* = 19).

In the next series of experiments, we determined if I_Na_ recovery from inactivation was altered in WT and MMRL1215 myocytes. Recovery was determined using a double pulse protocol and was evaluated at holding potentials of −120 mV and −100 mV for both cell types. Figure 7 shows representative traces recorded from a WT (Panel A) and MMRL1215 myocyte (Panel B) at a holding potential of −100 mV (protocol shown at the top of the figure). Reactivation was much faster when hp = −120 mV but was not significantly different between WT and MMRL1215. At hp = −120 mV, both cell types exhibited a fast and slow phase of recovery with time constants as follows: 6.7 ± 0.33 ms and 46.67 ± 6.69 ms for WT myocytes and 4.97 ± 1.10 ms and 35.18 ± 7.49 ms for MMRL 1215 myocytes (Figure 7C). Similarly, at hp = −100 mV the time constants for recovery were 18.5 ± 2.0 ms and 64.78 ± 10.78 ms for WT myocytes and 15.6 ± 2.70 ms and 69.77 ± 14.78 ms for MMRL 1215 myocytes (Figure 7D). 

**Figure 8:**
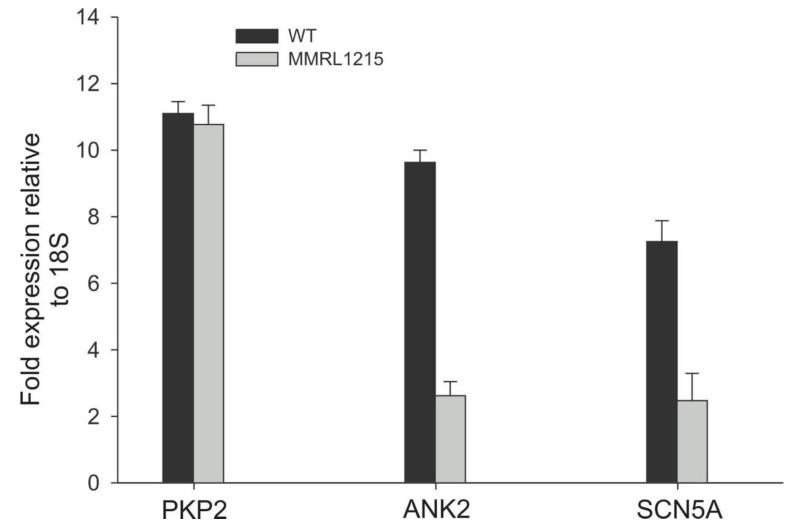
Expression profile of genes *PKP2*, *ANK2* and *SCN5A* obtained using real-time PCR.

RNA expression of Na^+^ channel α-subunits was examined by RT-PCR analysis. Since both *PKP2* and *ANK2* have been shown to associate with Nav1.5, we also examined message levels of both to determine if the mutations altered the expression. Our results show the lower I_Na_ density observed in hiPSC myocytes from MMRL1215 was paralleled by lower message of Nav1.5. Message levels of ANK2 were also significantly lower in MMRL1215 (Figure 8).

## 3. Discussion

### 3.1. Summary of Main Findings

In the present study, we identified a patient who exhibited an ECG consistent with signs of early repolarization syndrome coupled with a QT interval of 326 ms, suggesting a secondary diagnosis of short QT syndrome. Interestingly, these symptoms developed following high voltage electrocution. Genetic analysis of the patient showed the presence of multiple mutations in six genes of which only *ABCC9* was found to be associated with ERS [25]. However, none of the genetic variations have been associated with the secondary diagnosis of short QT syndrome. Using hiPSC-CMs derived from the patient, we found several abnormalities in EFP recordings, the Ca^2+^ transient magnitude, and a reduction in inward Na^+^ current. In addition, the patient-derived hiPSC-CMs showed shorter APDs which could be reversed with glibenclamide, suggesting ATP-sensitive K^+^ channels may be constitutively active in hiPSC-CMs from the patient. The reduction in Na^+^ current coupled with a constitutively active ATP-sensitive K^+^ channel may be the mechanism contributing to the mixed clinical phenotype.

The most striking observation in the study was the absence of a “pseudo” QRS complex in MMRL1215, suggesting a reduction in inward current(s). Voltage clamp analysis revealed a dramatic loss of I_Na_ but no change in I_Ca_. The loss in I_Na_ occurred even though no mutation in *SCN5A* was detected. Previous studies have demonstrated that mutations in *ANK2* and *PKP2* have been associated with ion channel dysfunction leading to cardiac arrhythmias. Mutations in *ANK2* are responsible for Long QT syndrome but these effects appear to be independent of any association with Nav1.5 [26]. Indeed, confocal analyses of WT and ankyrin-B-null mice ventricular myocytes revealed no difference in Na_v_1.5 immunolocalization suggesting no functional association of ankyrin-B and Nav1.5 [27]. Mutations in *PKP2* have been associated with a loss of I_Na_ resulting in BrS in a subset of patients. When BrS-associated mutation D26N in *PKP2* was transiently transfected into HL-1 cells, a reduction in I_Na_ by about 65% was noted [28]. Interestingly, our index patient (MMRL1215) carried the same D26N mutation in *PKP2*. Using hiPSC myocytes derived from the patient, we saw about a 60% reduction in I_Na_, strongly suggesting the loss of I_Na_ by D26N is responsible for primary diagnosis of ERS. 

Interestingly, this patient carried a number of genetic variations, but the clinical symptoms did not manifest until the patient was severely electrocuted. The mechanism behind this manifestation remains unclear. Previous investigations from our group have shown a similar scenario in the situation of acute myocardial infarction [29]. Results from those studies showed that the index patient carried a SCN5A mutation which remained subclinical, but expressed clinically after the patient had an acute myocardial infarction [29,30]. Upon recovery from the MI, multiple arrhythmias were observed suggesting that physiological stressors can unmask certain arrhythmias. The presence of several genetic variations may increase the propensity for developing arrhythmias. In the present study, the physiological stressor was electrocution which resulted in unmasking of the ERS/SQTS phenotype in this patient. In a retrospective study, high voltage electrocution has been shown to unmask or precipitate several cardiac arrhythmias which persisted for some time after the initial event [31]; the presence of several variations/mutations may increase the susceptibility of developing arrhythmias.

Numerous studies have modelled complex arrhythmia syndromes in hiPSC-CMs but with variable results. This variability has been attributed to many factors such as the methodology used to differentiate the hiPSC into the cardiac lineage as well as the maturity of pluripotent hiPSC-CMs. Compared to adult ventricular cells, hiPSC-CMs are deficient in several currents such as I_K1_ and I_to_ [32,33]. Despite these deficiencies, functional analysis of the patient-derived hiPSC-CMs in this study were dramatically different compared WT myocytes suggesting that MMRL1215 hiPSC-CMs were able to recapitulate this complexed ECG phenotype (such as ERS/SQTS).

The appearance of ERS is likely the result of a decrease in depolarizing inward I_Na_ during the early parts of the cardiac action potential. Alternatively, an increase in outward currents has been linked to the manifestation of ERS and SQTS [11]. The results of our study in hiPSC-CMs demonstrate that both mechanisms play a role. EFP recordings suggested a loss of inward current(s) and voltage clamp recordings confirmed I_Na_ was reduced. The loss of I_Na_ was dramatic although no mutation in *SCN5A* was noted. Previous studies have demonstrated that mutations in *PKP2* result in a loss of I_Na_ which is likely the situation in our patient. Similarly, the appearance of SQTS has been linked to mutations resulting in increased outward K^+^ currents and AP analysis confirmed that I_K,ATP_ appears to be constitutively active in MMRL1251 hiPSC-CMs. The presence of all variations (Table 1) may have an additive effect leading to the reduction of I_Na_ and an increase in I_K,ATP_ resulting in the clinical phenotype.

### 3.2. Conclusions

Many ventricular arrhythmia syndromes have been linked to mutations in *SCN5A*, *PKP2*, *ANK2*, and *ABCC9*. Interestingly, the index patient carried mutations/variations in these genes resulting in an overlap syndrome with characteristics of both ERS and SQTS. Although the patient carried six genetic variations of which three were predicted to be damaging by Sift analysis, the development of the clinical phenotype was not observed until the patient received a high voltage shock. Using hiPSC myocytes derived from the patient, we show clear functional alterations in a number of parameters such as altered EFP recordings, reduced I_Na_ density and altered action potential duration when compared to normal hiPSC myocytes. Our study shows that multiple genetic mutations can alter physiologic function and demonstrate the effectiveness of patient derived hIPSC myocytes to systematically examine the phenotypic changes. 

### 3.3. Limitations of the Study

All current recordings from the hiPSC-CMs were made after 21 days after plating. However, it is not certain hiPSC-CM kept in culture for longer time periods would result in changes such as a larger I_Na_ density or more mature calcium induced calcium release machinery such as development of t-tubules. Previous studies have shown that the biophysical and molecular characteristics of I_Na_ vary in adult ventricular, atrial and SA nodal myocytes [34,35,36]. Since hiPSC myocytes have been described as having nodal, atrial and ventricular phenotypes based on action potential morphology, we surmised that the 3 cell types may also show different I_Na_ densities. 

## 4. Methods

### 4.1. Subjects

IRB approval and informed consent was obtained from the patient included in this study. The proband was identified following repeated episodes of syncope and dizziness following electrocution. 

### 4.2. Next Generation Sequencing

An Ion Torrent Personal Genome Machine (PGM) was utilized to perform high throughput sequencing (HTS) which enabled the sequencing of 87 candidate genes at a time, a previously described [18]. These candidate genes encode ion channels and other proteins including pumps, exchangers, calcium handling proteins, gap junctional proteins, and structural proteins. They were selected based on their relative expression in human neuronal and cardiac tissue and their functional roles in generating action potentials and altering excitability. 

The Ion Torrent PGM (Life Technologies, Carlsbad, CA, USA) sequenced genomic DNA fragments generated through the use of Custom Ion Ampliseq 2.0 (Life Technologies, Carlsbad, CA, USA). The Coding Exons as well as intron borders for our 87 genes of interest from human genomic DNA were amplified through a massive multiplex PCR approach. DNA libraries were prepared by attaching adapters to these fragments, as well as a unique molecular barcode for each sample. Library quality was assessed using the Qubit 2.0 Fluorometer and the dsDNA HS Assay (Life Technologies, Carlsbad, CA, USA). An Ion Chef automation system then performed emulsion PCR, enrichment, and chip loading, followed by direct sequencing with the Ion Torrent PGM. 

### 4.3. Mutation Confirmation

All rare variations and mutations uncovered were confirmed using gold standard Sanger sequencing. PCR products were purified with a commercial enzyme (ExoSAP- IT, USB, Cleveland, OH, USA) and directly sequenced from both directions using Big Dye Terminator 3.1 chemistry on an Applied Biosystems 3730 DNA Analyzer (Life Technologies, Carlsbad, CA, USA). 

### 4.4. Bioinformatics Analysis

The Ion Torrent Suite software was used to map the sequencing reads to the DNA reference sequence [hg19] and identify variants through the VariantCaller plugin as well as the IonReporter analysis tool. Run Quality was assessed using the Coverage Analysis plugin which reports average depth of coverage, uniformity of coverage, and percent on target as well as other metrics. IonReporter compares all variations identified against NCBI’s dbSNP to rule out common SNP’s, as well as the 1000 genomes project and Exome Sequencing Project (ESP) to get published frequencies. Variants of interest were then flagged for verification through Sanger Sequencing. Mutations and rare variants were analyzed using several pathogenicity prediction tools such as PolyPhen2, SIFT and Grantham. When available, family members were sequenced for these mutations and rare variants to analyze the penetrance and establish a genotype–phenotype correlation.

### 4.5. hiPSC-CM Generation

Human WT iPSC (WiCell, Madison, WI, USA), passage 18–25, were maintained on growth factor-reduced Matrigel (Corning Corp., Corning, NY, USA.) coated plates in E8 medium (Gibco, Gaithersburg, MD, USA) with E8 supplement (Gibco, Gaithersburg, MD, USA). Patient derived human iPS cells were reprogrammed from fibroblasts with Oct4, Sox2, Lin28, and Nanog. A directed differentiation protocol to derive cardiomyocytes using serum-free, chemically defined media supplemented with CHIR99021, IWP2, Activin A, and KY0211 in stage specific manner, as previously described [37]. This protocol yielded contractile clusters by days 9 to 12 post-differentiation. Monolayers ranging between 20–50 days of maturity were plated on matrigel coated dishes and maintained with RPMI B27+ until use. 

### 4.6. Extracellular Field Potential and Impedance Recordings

Human iPSC-CMs were seeded and maintained on CardioExcyte 96 (Nanion Technologies, Livingston, NJ, USA) 96 well sensor plates. Extracellular field potential recordings were recorded with the CardioExcyte 96 when cells began to spontaneously contract (about 5–8 days after initial plating).

### 4.7. Electrophysiological Recordings

Voltage clamp recordings were made using patch pipettes fabricated from borosilicate glass capillaries (1.5 mm O.D., Fisher Scientific, Pittsburg, PA, USA). The pipettes were pulled using a gravity puller (Model PP-830, Narashige Corp., Amityville, NY, USA) and filled with pipette solution of the following composition (mM): For I_Ca_ recordings in hiPSC-CMs, the external solution contained (in mM): NaCl 140, KCl 5.4, MgCl_2_ 1, HEPES 10, D-Glucose 10, and CaCl_2_ 1.8, pH was adjusted to 7.4 with NaOH. The I_Ca_ patch pipette contained (in mM) CsCl 120, MgCl_2_ 1.0, EGTA 10, MgATP 5, HEPES 5, and CaCl_2_ 1.8, pH = 7.2 with CsOH [38]. For I_Na_ recordings in hiPSC-CMs, the external solution contained (in mM): N Methyl D-Glucamine 105, NaCl 40, CaCl_2_ 2.0, MgCl_2_ 1.0, glucose 10, HEPES free acid 10, CdCl_2_ 0.3, pH adjusted to 7.4 with NaOH/HCl. The pipette solution contained (in mM) NaCl 15, CsF 120, MgCl_2_ 1, KCl 5, HEPES 10, Na_2_ATP 4, and EGTA 10, pH adjusted to 7.2 with CsOH [39,40]. Current signals were recorded using a MultiClamp 700A amplifier (Molecular Devices, San Jose, CA, USA) and series resistance errors were reduced by about 60–70% with electronic compensation. Signals were acquired at 20–50 kHz (Digidata 1322, Molecular Devices, San Jose, CA, USA) and analyzed with a microcomputer running pClamp 9 software (Molecular Devices, San Jose, CA, USA). All patch recordings were made at 37 °C with the exception of I_Na_ which was performed at room temperature.

Sharp microelectrodes (40–60 MΩ) filled with 2.7 M KCl were used to record APs from spontaneously beating monolayers, as previously described [40]. Monolayers were superfused with HEPES-Tyrode’s solution of the following composition (in mM): NaCl 140, KCl 5.4, MgCl_2_ 1, HEPES 10, D-Glucose 10, and CaCl_2_ 1.8, pH was adjusted to 7.4 with NaOH. The microelectrodes were connected to an MultiClamp 700B amplifier (Molecular Devices, San Jose, CA, USA) operating in current clamp mode. All signals were digitized (sampling rate = 50 kHz), stored on a computer and analyzed using pClamp9 acquisition suite (Molecular Devices, San Jose, CA, USA).

### 4.8. Fluorescence Imaging

Confocal Ca^2+^ imaging experiments were performed with an Olympus Fluoview laser-scanning confocal microscope (Olympus Life Science, Center Valley, PA, USA) as previously described [18,41]. Fluo 4-AM [dissolved in 20% F-127 pluronic in dimethyl sulfoxide (DMSO), final concentration 15 µM] was added to hiPSC-CMs and incubated for 20 min at room temperature. Fluo-4 loaded hiPSC-CMs were placed in a perfusion chamber and excited at 488 nm using an argon laser, and fluorescence emission was detected via a 520-nm band-pass filter and photomultiplier tube. Confocal images were acquired with the Fluoview acquisition software program and spontaneous activity was recorded on a personal computer for later analysis. Images acquired with Fluoview acquisition software were analyzed with ImageJ and Fluoview analysis software (Olympus Life Science, Center Valley, PA, USA).

### 4.9. RNA Isolation and Quantitative Real-Time PCR

Quantitative real-time PCR was performed as described previously [37]. RNA was isolated from hiPSC cardiomyocytes 15 days after plating using the TRiZOL reagent (Life Technologies, Carlsbad, CA, USA), according to the manufacturer’s protocols. A NanoDrop^®^ spectrophotometer (Thermo Fisher, Wilmington, DE, USA) was used to quantify RNA. Superscript™ III Reverse Transcriptase kit (Life Technologies, Carlsbad, CA, USA) approach was used to synthesize cDNA from 1–5 μg of RNA. Standard curves were generated to calculate the efficiency of the probe.

TaqMan Probe sets:

SCN5A—Hs00165693_m1 (Life Technologies)
PKP2—Hs00428040 m1 (Life Technologies)
ANK2—00153998 m1 (Life Technologies)
18s-Hs03003631_g1 (Life Technologies)

All no-template control wells were negative in all PCR runs. The mRNA levels were referenced to 18s and the ΔΔCt relative quantification method was used [19].

### 4.10. Statistical Analysis

All data are presented as Mean ± SEM. Statistical comparisons were made using ANOVA followed by a Student–Newman–Kuels, or Student’s *t*-test, as appropriate. Significance was determined at *p* < 0.05.

## Figures and Tables

**Table 1:** MMRL1215 87 gene panel results.

# Locus	Gene	Exon	Protein	Coding	Sift	Dbsnp
chr1:237947931	*RYR2*	90	p.Arg4307Cys in Ryanodine receptor 2	c.12919C>T	Tolerated	rs200092869
chr4:114286207	*ANK2*	41	p.Val3634Asp in Ankyrin-B	c.10901T>A	Damaging	rs66785829
chr12:5154064	*KCNA5*	1	p.Ala251Thr in Kv1.5	c.751G>A	Tolerated	rs12720442
chr12:22035732	*ABCC9*	14	p.Arg663Cys in ATP Binding Cassette C9	c.1987C>T	Damaging	rs200349671
chr12:33049590	*PKP2*	1	p.Asp26Asn in Plakophilin 2	c.76G>A	Damaging	rs143004808
chr19:615946	*HCN2*	8	p.Pro715_Pro717del in Hyperpolarization activated Cyclic Nucleotide channel 2	c.2156_2164delCGCCGCCGC		rs879255365

**Table 2:** Electrophysiological Parameters of WT and MMRL1215 hiPSC-CMs.

	Spontaneous Cycle Length (bpm)	AP Amplitude (mV)	APD50 (ms)	APD90 (ms)	Vmax (V/s)	Maximum Diastolic Potential (mV)
**CONTROL**	61.9 ± 4.2 (*n* = 14)	105.7 ± 4.4 (*n* = 14)	380.6 ± 31.9 (*n* = 14)	456.2 ± 29.4 (*n* = 14)	26.9 ± 2.8 (*n* = 14)	−72.5 ± 1.6 (*n* = 14)
**MMRL1215**	48.7 ± 4.0 (*n* = 16) *	102 ± 2.2 (*n* = 16)	206.6 ± 33.8 (*n* = 16) *	267.5 ± 38.6 (*n* = 16) *	22.6 ± 3.3 (*n* = 16)	−71.9 ± 1.4 (*n* = 16)

* Significantly different from control (*p* < 0.05).

**Table 3:** Effect of Glybenclamide (10 µM) on WT and MMRL1215 hiPSC-CM Electrophysiological Parameters.

	Spontaneous Cycle Length (bpm)	AP Amplitude (mV)	APD50 (ms)	APD90 (ms)	Vmax V/s)	Maximum Diastolic Potential (mV)
MMRL1215 –glybenclamide	43.3 ± 4.8 (*n* = 6)	100.2 ± 5.0 (*n* = 6)	240.9 ± 42.8 (*n* = 6)	295.7 ± 44.4 (*n* = 6)	33.4 ± 10.9 (*n* = 6)	−67.1 ± 2.8 (*n* = 6)
MMRL1215 +glybenclamide	54.3 ± 12.3 (*n* = 6)	91.3 ± 10.4 (*n* = 6)	262.5 ± 33.8 (*n* = 6)	403.5 ± 50.6 (*n* = 6) *	24.8 ± 10.5 (*n* = 6)	−61.9 ± 5.7 (*n* = 6)
WT –glybenclamide	56.2 ± 5.7 (*n* = 10)	105.4 ± 1.7 (*n* = 10)	354.69 ± 31.9 (*n* = 10)	464.7 ± 34.3 (*n* = 10)	29.4 ± 3.4 (*n* = 10)	−67.1 ± 2.8 (*n* = 10)
WT +glybenclamide	51.4 ± 9.1 (*n* = 10)	105.5 ± 3.5 (*n* = 10)	316.3 ± 35.0 (*n* = 10)	424.2 ± 34.3 (*n* = 10)	31.0 ± 4.8 (*n* = 10)	−61.9 ± 5.7 (*n* = 10)

* Significantly different from –glybenclamide (*p* < 0.05).

**Table 4:** Parameters of Calcium Transients from WT and MMRL 1215 hiPSC-CMs.

	Spontaneous Cycle Length	Number with Regular Spontaneous Cycle Length	% with Regular Spontaneous Cycle Length	(F-Fo)/Fo
**CONTROL**	1410.1 ± 42.3 ms (*n* = 77)	77/78	98.7%	5.76 ± 0.36 (*n* = 77)
**MMRL 1215**	1460.0 ± 58.0 ms (*n* = 82)	82/86	95.3%	4.81 ± 0.36 (*n* = 82) *

* Significantly different from control (*p* < 0.05).

## Data Availability

The data presented in this study are available on request from the corresponding author at jcordeiro@mmri.edu. The data are not publicly available due to Masonic Medical Research Institute confidentiality policies.
